# Perception of Undergraduates in the Field of Periodontology

**DOI:** 10.7759/cureus.48308

**Published:** 2023-11-05

**Authors:** Aachal Lande, Khushboo Durge, Shivani Rawat, Amit Reche, Prasanna R Sonar

**Affiliations:** 1 Dentistry, Sharad Pawar Dental College and Hospital, Datta Meghe Institute of Higher Education and Research, Wardha, IND; 2 Periodontics, Sharad Pawar Dental College and Hospital, Datta Meghe Institute of Higher Education and Research, Wardha, IND; 3 Public Health Dentistry, Sharad Pawar Dental College and Hospital, Datta Meghe Institute of Higher Education and Research, Wardha, IND; 4 Oral Medicine and Radiology, Sharad Pawar Dental College and Hospital, Datta Meghe Institute of Higher Education and Research, Wardha, IND

**Keywords:** career, undergraduates, scope, periodontal ligament, periodontology, periodontium

## Abstract

The dentistry specialty known as periodontology focuses on problems with the tissues that support and surround teeth. A thorough understanding of basic sciences and health care is necessary for periodontology. To ensure that the dentition has adequate form, function, esthetics, and comfort, it is essential to have a thorough understanding of the relationship between periodontal tissues and how it relates to other branches of dentistry, such as restorative dentistry, orthodontics, prosthodontics, and implants. The periodontal ligament is a soft tissue between the alveolar bone and teeth. Since the alveolar bone is the foundation for successful dental treatment, proceeding with subsequent dental procedures, including restorative dentistry, implants, and prosthetic uses, is only possible with sufficient bone support. The current review article aims to assess the perception of undergraduate students regarding periodontics and its range of practice. It also gives a general overview of the field of periodontology by outlining current practices, exploring how students perceive this branch's range of courses, and emphasizing more recent and sophisticated developments.

## Introduction and background

The relationship between calculus and periodontal disease was often explored, and it was frequently considered that underlying systemic diseases caused periodontal illnesses. The cementum, alveolar bone, gingiva, and periodontal ligament comprise the complex structure known as the periodontium. They safeguard the underlying systems and enable the teeth to connect to the bone. Most diseases, with periodontal disease being the most common of them all, were present in the embalmed bodies of ancient Egyptians. The fact that the topic was covered in old medical and surgical publications is surprising. The Ebers papyrus provides multiple suggestions for bolstering the teeth and gums and numerous delusions about gingival disease. These remedies were applied to the gums as a paste made from honey, vegetable gum, leftover beer, or other materials from various plants and minerals.

Even though periodontology only recently underwent institutionalization in the early 20th century, periodontal disease and its treatment have long been understood. For a very long time, periodontal disease was thought to be caused by the buildup of calculus or tartar. Many prehistoric societies were aware of periodontal conditions and their therapies. Since ancient times, the tooth has carved out a wonderful niche in the study of the human body, and around that also revolves periodontology, the study of gums. Gingival and periodontal illnesses have affected people since the dawn of time, and research in the field of paleopathology has revealed that early man was impacted by destructive periodontal disease, which is indicated by bone loss in ancient cultures such as ancient Egypt and early Pre-Columbian America [1]. By emphasizing the value of this specialty in clinics, periodontists should work to improve the standards of the field as a whole. First, one should assess the significance of the period-systemic relationship, or periodontitis' connection to conditions such as diabetes mellitus, cardiovascular disease, pregnancy, and stress. Periodontics includes a variety of scientific disciplines. Periodontics has developed over time to include a variety of scientific fields. In this article, periodontology is mostly discussed in terms of its conventional diagnostic tools and treatments, followed by more recent and complex developments.

Calculus and periodontal disease were frequently linked, and periodontal diseases were often thought to result from underlying systemic problems [2,3]. When John W. Riggs, now famous for coining the phrase "Riggs disease," began limiting his practice to treating illnesses of the periodontium, periodontics as a field of study officially got underway in the middle of the 19th century [2,4].

Methodology

**Figure 1:**
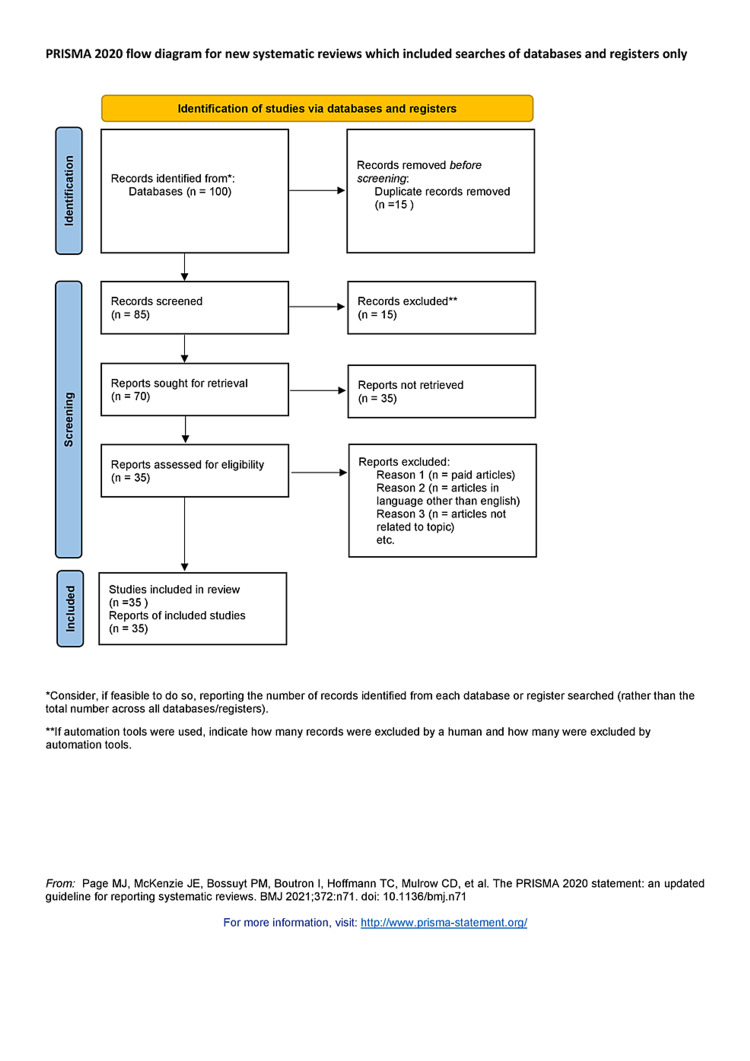
PRISMA flow diagram for search strategy PRISMA, Preferred Reporting Items for Systematic Reviews and Meta-Analyses

We conducted a systematic search through PubMed and Google Scholar in September 2022 using keywords such as "Periodontology," "Scope," "Undergraduates," ((Periodontology[Title/Abstract]) OR ("Periodontology"[MeSH Terms]),(("Scope"[Title/Abstract]) OR ("Scope"[MeSH Terms]),("Undergraduates[Title/Abstract]) OR ("Undergraduates"[MeSH Terms]). Finally, a total of 35 articles were included. Figure 1 shows the PRISMA flow diagram for the search strategy.

## Review

Ancient civilizations

Aulus Cornelius Celsus, a Roman encyclopedist known best for his medical writings in De Medicina, also wrote about periodontal disease. He described it as a disease that affects the soft parts of the mouth. He describes the treatment as “if the gums separate from the teeth, it is beneficial to chew purslane, pears, and apples and keep their juices in the mouth” [5]. Periodontal disease dates back to 5000 BC; The Royal Library of Ashurbanipal has evidence from clay tablets. Early civilizations used myrrh, asafetida, opopanax, and pine-turpentine to treat loose teeth. Hippocrates believed it was caused by calculus accumulation, causing gums to detach from teeth and causing bad smells.

1800s Periodontal Disease

Destructive gum disease, primarily systemic or local, was controversial in the 19th century regarding its causation and treatment. After American dentist John W. Riggs, periodontal disease, sometimes known as Riggs' disease, was named [6]. In his short novella Happy Memories of the Dental Chair, Mark Twain discusses his periodontal therapy and how Dr. Riggs told him that most people, even those with perfect teeth, have Rigg's disease [7].

1900s Periodontal Disease

Gillette Hayden and Grace Rogers Spalding, two female dentists, spearheaded the founding of the first national association devoted to the detection and management of periodontal disease in May 1914. The American Academy of Oral Prophylaxis and Periodontology was the organization in question. It was renamed the American Academy of Periodontology in 1919.

Periodontics: 21st century and the future

In the 21st century, periodontists and dental hygienists play a crucial role in patient care due to the oral-systemic link. The risk of some systemic disorders is raised by periodontal disease [8]. Recent research has identified inflammation and bone loss as potential treatment targets for periodontal disease. Dental hygienists must push for more autonomy and focus on the future. Due to their growing popularity, dental implants are recognized as one of the main therapeutic alternatives for patients needing to replace missing dentition. Over the past few years, dental implant placement has gained popularity, and more clinical settings are now utilizing implants. Dental implants are routinely placed in patients who have advanced periodontitis. According to certain accounts, the use of implants in patients who are partially or completely edentulous and at risk for periodontitis is commonly disputed. Besides this, radiographs have been valuable for many years in diagnosing periodontal disease and evaluating treatment effects. Besides various image reconstruction procedures such as tomosynthesis, computer-based image acquisition and processing techniques have enhanced the utilization of radiodiagnosis in periodontics. Advances in different biochemical and microbiological diagnostic methods have also been beneficial in diagnosing periodontal diseases.

In periodontics, occlusion is crucial. Forces acting in one direction might induce the tooth to tip in the opposite direction or cause the tooth to move parallel to the force, creating "bodily movement" zones of compression and tension in the periodontal ligament. There are zones of stress, which cause resorption to rise. Similarly, occlusion-related trauma can arise from two different mechanisms: either the tissue-adaptive threshold is compromised to such an extent that the residual supporting tissues are incapable of withstanding the physiological occlusal forces, or the intensity of forces exceeds the adaptive threshold of the periodontal supporting tissues (primary trauma from occlusion).

Scope of periodontics in dentistry

A dynamic field, periodontics encompasses complicated treatment planning and a wide range of therapeutic methods, ranging from basic to cutting-edge therapies. It is a comprehensive specialty that offers chances in academic clinical practice supported by solid scientific information and is based on evidence, paving the way for future research horizons. Everything else in dentistry revolves around periodontics since it maintains the health of the teeth so that other treatments can be provided. It is a special area of dentistry that allows for both tooth preservation and implant-based tooth replacement.

Where are we lagging?

The referral mechanism used by general practitioners and other co-professionals in other specialties is one area where periodontology as a specialty needs to catch up. There may be several causes. The proliferation of several dental institutions is primarily to blame for the appearance of undergraduates who are unprepared and lacking in knowledge and experience. They struggle to identify periodontal disease; even when they do, they only perform the non-surgical portion, delaying or avoiding the surgical part. The patient is not recommended by a periodontist [9]. Furthermore, BDS (Bachelor of Dental Surgery) graduates must be given detailed instructions on when to send a patient to a periodontist [10]. Therefore, we must put in some effort and education in this area. To preserve the reputation of the periodontist as the person who saved the teeth, we must work extra hard in this area and teach our students when it is best to refer the patient. In our circumstances, dental insurance is still in its infancy. Thus, individuals are hesitant to see a specialist.

The field of periodontics encompasses many other dental specialties, including forensics, prosthodontics, conservative dentistry, endodontics, and orthodontics. Over the years, periodontics has expanded to encompass many scientific fields.

Why do undergraduates hate or dislike periodontology?

It is believed that dental professionals, in particular, must have knowledge of and attitudes about oral health to promote awareness of and access to oral health treatment effectively. The general public, dental students during their education, and dental professionals all exhibit a wide range of attitudes toward oral health care methods. The ability of dental students to translate knowledge into action at the community level to promote excellent oral health during their education and after graduation depends directly on their oral health knowledge, attitude, and practice [11]. These attitudes are a natural reflection of the students' experiences, methods of instruction and learning, use of social media, impressions of their culture, ideas held by their families, and other real-world events they encountered while in school. Because of the impact of the student's academic standing and the lack of properly channeled assessment study through the courses as per the curriculum, BDS students are not exposed to clinical scenarios and practices during the first and second years of their academic careers [12]. They also frequently overlook and disregard the importance of understanding personal oral care practices.

More information about undergraduate dentistry students' attitudes, knowledge, and self-awareness who have clinical experience is needed. Therefore, the current survey was initiated to assess undergraduate non-clinical dental students' understanding of periodontics, a dental specialty concerned with the gingival and periodontal tissues supporting the teeth, to ascertain their oral health, behavioral attitude, and self-awareness. Aspiring dental students during their early academic lives need more self-awareness, information, and perspective about many gingival and periodontal disease elements, ranging from oral health care practices to higher prevention and treatment techniques. Despite having good knowledge of periodontics (86%), only 39% of BDS non-clinical students consistently practice good oral hygiene, and only 72% acknowledge that scaling and root planing are required to treat periodontal diseases. In addition, 44% students needed to be made aware of the various treatments and clinical procedures used. Indeed, how dental students conduct themselves toward patients once they begin working in clinical settings will greatly influence their ability to set a positive example for others by practicing and promoting oral health. Therefore, efforts must be made to integrate useful knowledge by developing problem-based learning environments into the undergraduate BDS curriculum of non-clinical students.

Scope of periodontics among undergraduates

In addition to treating periodontium, periodontology promotes the health of supporting structures and facilitates the effective operation of hard systems. The scope of practice in periodontics greatly expands into other dental disciplines. Periodontists have many career possibilities that enable them to lead secure and satisfying work lives. Working in periodontics clinical practice, which includes a range of practice settings such as regular practice or specialized practice in dentistry clinics, polyclinics, and multispecialty clinical settings, may be one of these alternatives.

Second, a periodontist can work in a relaxing and secure setting because they are eligible for several government positions. Teaching undergraduate and graduate students at public and private dental institutions is another aspect of an academic career. From assistant professor to full-time chair, people will progress through various stages in their academic careers, which may be a gratifying and fulfilling experience.

**Figure 2:**
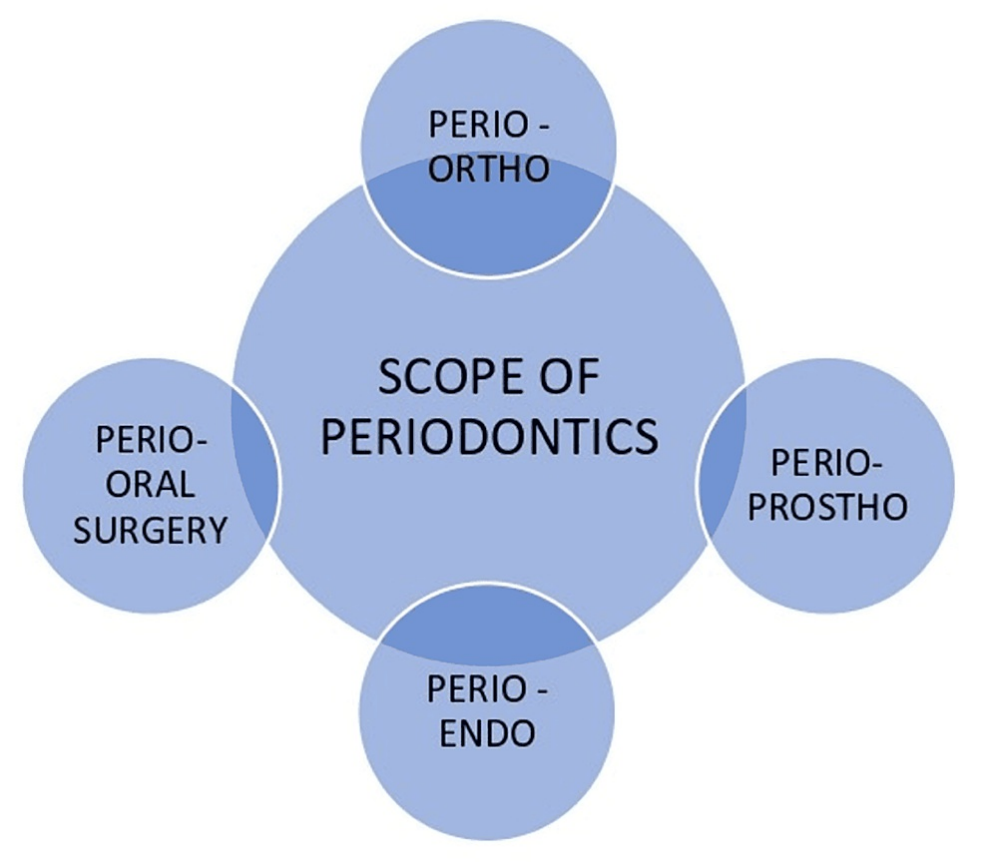
Scope of periodontics among various branches of dentistry Image Credit: Aachal Lande

Furthermore, periodontal research always updates its guiding principles and plans new ideas. The initial focus of periodontics research is part of the postgraduate program. Many local, national, and international organizations provide assistance in this field as well as funds to develop a research career. Careers and training in this subject are offered by governmental agencies, academic institutions, and private company research institutes [13]. The field of periodontics considerably broadens its scope to include other dental specialties, as represented in Figure 2.

Perio-ortho interaction

Since the periodontal ligament, an essential component of the periodontium, acts as the mechanism for orthodontic tooth movement, it is impossible to overstate the significance of the effects of orthodontic treatment on periodontology. In patients with compromised periodontal health, special care must be taken to guarantee that orthodontic treatment is performed on healthy periodontal tissues. Another area where the ideas of these two specialties collide is in the notion of "periodontally accelerated osteogenic orthodontics," which accelerates orthodontic tooth movement by combining particulate bone grafting and selective alveolar corticotomy with orthodontic therapy [14]. Temporary anchorage devices, or mini-implants, are routinely used for orthodontic anchorage. Since orthodontic therapy in adults has evolved into a common and reliable treatment viewpoint, many orthodontic patients arrive with some degree of periodontal disease or a diminished periodontium due to a history of periodontitis [15,16]. When a tooth is positioned improperly or when pathological migration occurs, the result might be a persistent periodontal infection. Supplemental orthodontic treatment may be important in creating the ideal foundation for restoring an aesthetically pleasing and functioning dentition [17]. Additional orthodontic recommendations for specific periodontal disease manifestations include the extrusion of elongated teeth, orthodontic extrusion for treating infra-osseous flaws or increasing soft and hard tissue surrounding hopeless teeth before implant insertion can close spaces, straightening overly tipped molars, and treating infra-osseous defects [18-20].

Perio-prostho interaction

Replacing missing or damaged teeth with prostheses is necessary to achieve the desired prosthetic rehabilitation lifetime and enhance patients' comfort, function, health, and appearance [21]. The multidisciplinary perio-prostho approach to periodontal health includes the long-term upkeep of prosthetic repair and inflammation management. Prosthesis cleanability and biological width are crucial when planning and carrying out prosthodontic treatment. How quickly it can be cleaned depends on the prosthesis's design, the prosthesis's anatomy, and the patient's cooperation. The periodontium's robust framework and restorations with periodontal-friendly methods are essential for the success of any prosthodontic therapy.

Perio-endo interaction

Because they may be challenging to diagnose and treat, coupled endodontic-periodontal lesions are the principal source of the observed interaction between periodontology and endodontics. Tooth pulp and periodontium can communicate through the apical foramen, small exit portals, and exposed dentinal tubules. The apical foramen and the apertures of the microscopic outlets of departure, where the infected pulp is located, stimulate the periodontal tissues' inflammatory response [22]. The pulp may not be severely affected by periodontal disease unless it results in extensive attachment loss; in this case, lateral or accessory canals may be connected to the oral environment. The dental pulp may induce an ongoing inflammatory response, resulting in pulp necrosis when the apical foramen is impacted [23,24]. Microbiologically, common pathogen profiles have been found in pulp chamber samples and teeth with periodontitis in chronic conditions. These infections are thought to cause pulp necrosis and the development of inflammatory periapical lesions [25,26]. Additional contributing factors, including poor endodontic therapy or restoration, trauma, root resorption, and dental anomalies, which lower treatment success rates, have affected the biological equilibrium between the endodontic and the surrounding periodontal tissues [27-29].

Awareness of career opportunities among undergraduates

**Figure 3:**
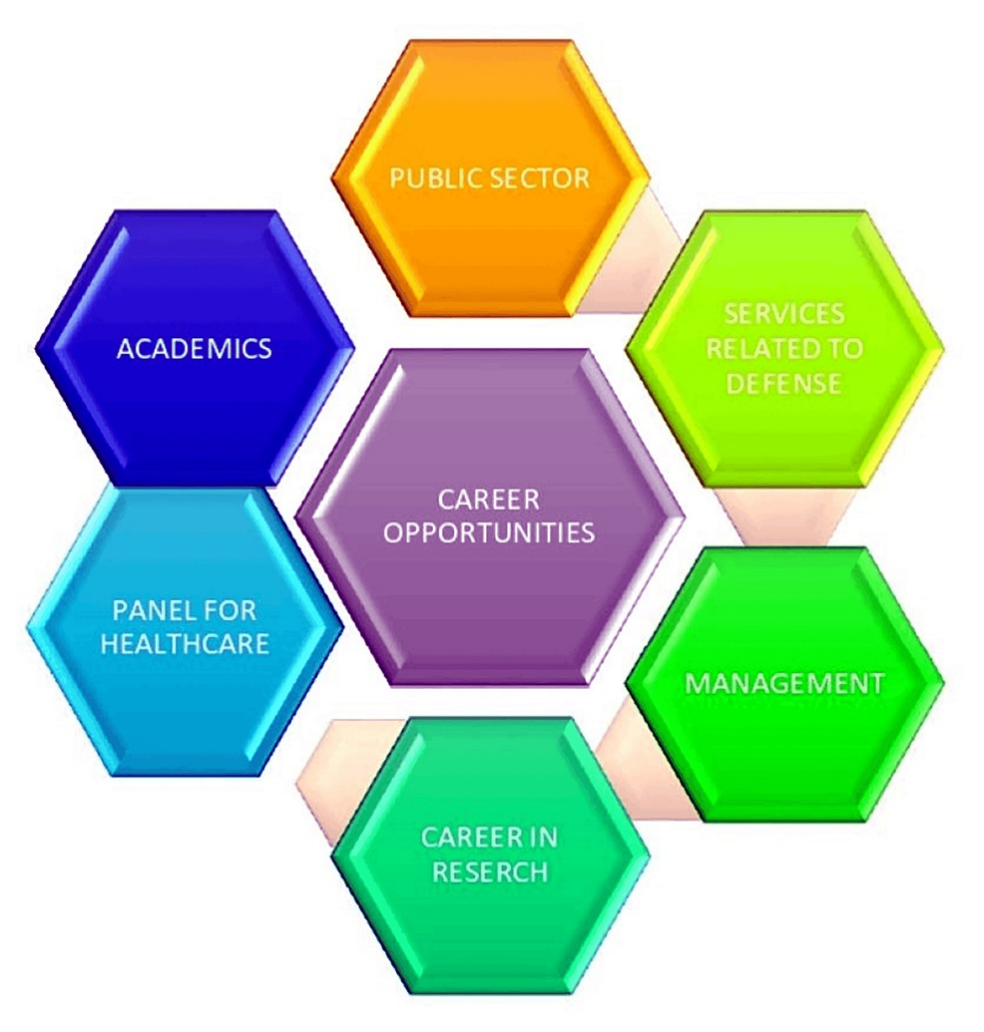
Career opportunities among undergraduates Image Credit: Aachal Lande

There are many and varied career choices in periodontics, allowing a periodontist to have a fulfilling and secure existence. Figure 3 shows the career opportunities among undergraduates.

Public Sector

A periodontist can work in a welcoming and safe environment because they are qualified for various jobs in the worldwide public sector. The Indian government promotes dentists since there are many career opportunities in both urban and rural locations And also provides employment options in a variety of industries.

Services Related to Defense

The army, navy, and air force all have specialty positions assigned by the defense/armed forces, which is a highly structured force. Serving the country as a dental officer is an honor and entails several excellent rewards that can help secure one's financial future. Along with financial incentives, placements in defense services also offer low-cost or free health and life insurance, access to retirement plans, housing allowances, and paid vacations. A serving periodontist officer would have the exclusive right to promote periodontal health in a way that indirectly improves overall health.

Academics

Teaching undergraduate and graduate students is part of an academic career in public and private dentistry schools. From assistant professor to full-time chair, people will rise through numerous stages in their academic careers. The completion of a career in academia can be pleasant and rewarding.

Dental Product Advice

Another career option is working in the research and advisory divisions of pharmaceuticals and different businesses that produce goods for oral health, biomaterials used in periodontics, and items related to oral health.

Panel For Health Care

The central and state government schemes are the gold standard for providing health care for central government employees and certificates that may require further education. There are licensing examinations in some countries that recognize the degree, allowing students to begin working immediately.

Academics: Continuing Education

A doctor of philosophy in periodontics degree provides students with various classroom and clinical research experiences designed to help them comprehend certain aspects of the profession. Numerous institutes in India and abroad offer a comprehensive range of fellowship and postgraduate courses in lasers, implantology, cosmetic dentistry, and regenerative medicine. Periodontists can always use these programs to gain more experience.

Career in Research

Oral and maxillofacial research now provides a wealth of options owing to the exceptional age of discovery we are living in. The principles of the past are continually being revised, and new concepts are being planned in periodontal research. The postgraduate curriculum served as the foundation for periodontics. This has to be advanced, which can be made possible with joint research. Many domestic and international organizations offer support in this area and money to pursue a research career. Governmental organizations, academic institutions, and private company research facilities all offer employment and training opportunities [30].

The future of periodontics

More promising treatments are on the horizon, notwithstanding the great advancements in periodontal therapy that have been observed recently. Many developments in oral health care over the years have had a big impact on periodontics practice. The fact that periodontology is a dynamic science that constantly adapts and refocuses on the basis of new research covering a wide range of scientific endeavors is one of its greatest assets.

Expanding the scope of lasers and implants and their significance among undergraduate students

Periodontal history must be evaluated when dental implants are advised for missing teeth. Although dental implants are widely employed in these situations, the peri-implant tissues around them are still vulnerable to the same host-modulated, plaque-induced variables that lead to periodontal inflammation [31]. Remaining pockets have been noted to serve as potential infection hotspots for dental implants, and putative periodontal bacteria have been found to persist even after infected teeth have been extracted [32]. Clinical studies suggest that placing dental implants in chronic or aggressive periodontitis patients may lead to deteriorating clinical outcomes, including survival rates and marginal bone loss [33]. Findings from recent, well-planned systematic reviews lend credence to this.

Based on a risk assessment, a supportive, preventative approach is necessary for all populations. Dental implants can be suggested to patients who have successfully undergone treatment for chronic and aggressive periodontitis, even though a history of periodontitis, even the severe varieties, may not be regarded as an absolute contraindication for implant therapy. Patients should be strongly encouraged to adhere to plaque control and restrict risk factors, such as smoking behaviors, common for periodontal inflammation and peri-implantitis, throughout long-term and specialist supportive care.

Challenges and limitations

Since they only receive manual scaling as clinical experience during their undergraduate years, it is sad that most undergraduate students think that periodontics could be more varied and insipid. They rarely get to see or participate in difficult periodontal surgical operations and frequently need to gain knowledge of interdisciplinary periodontics [34]. Dentists have largely taken over the responsibility for providing periodontal care due to contemporary technologies and treatments becoming simpler. This phenomenon is termed the "innovator's dilemma" and was first noted by Christensen [35]. Most of a periodontist's specialist work is handled by a dentist who has acquired some knowledge by taking some fundamental courses, which puts them in an unusual situation. The expansion of dental clinics in cities would worsen matters for a periodontist because people prefer to go to the nearest dentist.

## Conclusions

The future of the field of periodontics is promising and lucrative, but it will require a lot of work and obstacles. Although periodontics appears to have a bright future, this will likely happen with a lot of work and obstacles. Finding employment for everyone is becoming more and more of a struggle as more postgraduate periodontists graduate each year. Financial insufficiency is another significant hurdle preventing a periodontist from opening their clinic or purchasing the necessary equipment. One of its biggest advantages is that periodontics is a dynamic topic that frequently shifts and refocuses in reaction to discoveries and breakthroughs in contemporary study in neighboring domains. Thanks to the paradigm shift in treatment choices and the addition of implantology as a subspecialty, periodontists will have plenty of employment alternatives. It will occur after the beginning rather than the beginning of the end.
